# Successful laparoscopic management of strangulated left paraduodenal hernia: A case report and review of minimally invasive approaches

**DOI:** 10.1016/j.ijscr.2024.110566

**Published:** 2024-11-07

**Authors:** Hariruk Yodying

**Affiliations:** Department of Surgery, HRH Princess MahaChakri Sirindhorn Medical Center, Faculty of Medicine, Srinakharinwirot University, 62 Ongkharak, Nakhon Nayok 26120, Thailand

**Keywords:** Paraduodenal hernia, Laparoscopic surgery, Bowel strangulation, Indocyanine green imaging, Bowel ischemia

## Abstract

**Introduction:**

Paraduodenal hernias are rare congenital anomalies that can lead to acute bowel obstruction and strangulation. Laparoscopic management of these complex cases in emergency settings remains challenging, particularly when bowel ischemia is present.

**Case presentation:**

We report a case of a 56-year-old woman presenting with acute small bowel obstruction due to a strangulated left paraduodenal hernia. Emergency laparoscopic surgery revealed ischemic bowel segments within the hernia sac. We utilized indocyanine green (ICG) fluorescence imaging to assess bowel perfusion intraoperatively, guiding our decision for bowel resection. The procedure involved hernia reduction, resection of non-viable bowel, and primary anastomosis, followed by hernia defect closure. Despite encountering a small bowel injury during reduction, we successfully completed the procedure laparoscopically.

**Discussion:**

This case demonstrates the feasibility of laparoscopic management for complicated paraduodenal hernias with bowel strangulation in emergency settings. The use of ICG imaging for real-time perfusion assessment represented a novel application in this context, aiding in the precise identification of ischemic bowel segments requiring resection.

**Conclusion:**

Laparoscopic repair of strangulated paraduodenal hernias is feasible and effective, even in emergency scenarios. The integration of advanced imaging techniques like ICG fluorescence may enhance intraoperative decision-making, particularly in assessing bowel viability. This approach potentially reduces the extent of bowel resection and improves outcomes in these complex cases.

## Highlights

• Rare case of strangulated left paraduodenal hernia managed laparoscopically.
• Use of ICG fluorescence imaging to assess bowel viability.
• Successful bowel resection and primary anastomosis performed laparoscopically.
• Demonstrates feasibility of minimally invasive approach in emergency settings.
• Contributes to limited literature on laparoscopic repair of strangulated internal hernias.

## 1 Introduction

Paraduodenal hernias, while rare, represent a significant surgical challenge, particularly when presenting with acute bowel obstruction and strangulation. These congenital anomalies arise from errors in the embryological rotational development of the midgut. Despite accounting for only 0.2–0.9% of all hernias, they constitute 30–53% of internal hernias, making them the most common type of congenital internal hernia [1,2]. Left-sided paraduodenal hernias, comprising approximately 75% of cases, are more prevalent than their right-sided counterparts [3].

The clinical presentation of paraduodenal hernias poses a significant diagnostic challenge for clinicians. Symptoms range from chronic, vague abdominal discomfort to acute small bowel obstruction with the potential for strangulation [4,5]. This variability in presentation, coupled with the rarity of the condition, often leads to delayed diagnosis or misdiagnosis. Consequently, paraduodenal hernias carry a substantial risk of complications, with reported mortality rates of 20–50% in cases presenting with acute bowel strangulation [6].

Advances in imaging techniques, particularly computed tomography (CT), have improved the preoperative diagnosis of paraduodenal hernias [7]. However, the definitive diagnosis and assessment of bowel viability are frequently made intraoperatively.

The management of paraduodenal hernias has evolved significantly, with laparoscopic approaches gaining traction [8]. While laparoscopic repair offers benefits such as reduced postoperative pain and faster recovery, technical challenges persist, especially in emergency settings with bowel strangulation.

This case report presents a unique instance of emergency laparoscopic management of a complicated left paraduodenal hernia with bowel strangulation. We highlight the feasibility of minimally invasive approaches in acute settings and describe the application of indocyanine green (ICG) imaging for real-time assessment of bowel perfusion. This case report has been reported in line with the SCARE 2023 criteria [9].

## 2 Case presentation

A 56-year-old woman presented to the emergency department with a one-day history of acute onset, increasingly severe colicky abdominal pain accompanied by bilious vomiting. The patient had no significant past medical history and no previous abdominal surgeries. On examination, her vital signs were: blood pressure 110/70 mmHg, heart rate 92 beats/min, respiratory rate 18 breaths/min, and temperature 37.2 °C. Her BMI was 24 kg/m^2^.

Physical examination revealed a dehydrated patient with abdominal distension, diffuse tenderness, and guarding. Laboratory findings were significant for leukocytosis (white blood cell count 14,500/μL), indicating an inflammatory process and raising suspicion for potential bowel ischemia.

**Fig. 1:**
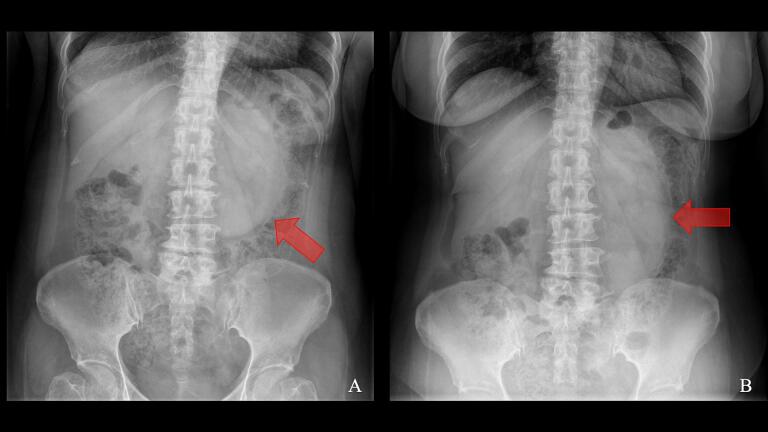
Abdominal X-ray findings in left paraduodenal hernia. A: Supine view, B: Upright view. Abdominal X-ray demonstrating a circumscribed soft tissue mass in the upper quadrant (arrows), displacing the transverse colon inferiorly.

**Fig. 2:**
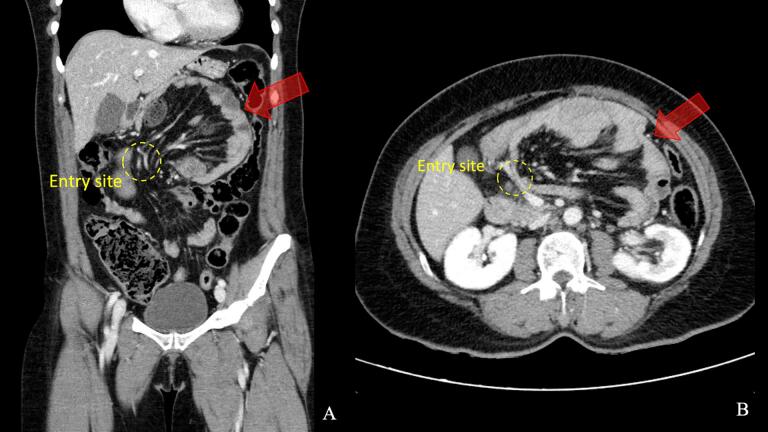
CT scan characteristics of left paraduodenal hernia. A: Coronal view, B: Axial view. Contrast-enhanced CT scan showing a cluster of small bowel loops (arrows) situated between the stomach and pancreas, with associated mass effect.

Initial diagnostic imaging included a plain abdominal X-ray, which demonstrated a circumscribed soft tissue mass in the upper quadrant, displacing the transverse colon inferiorly (Fig. 1). Given the concerning clinical picture suggestive of bowel obstruction, we performed a contrast-enhanced computed tomography (CT) scan of the abdomen. The CT revealed an abnormal cluster of dilated small bowel loops with wall thickening, situated between the stomach and pancreas. This mass effect caused superior displacement of the posterior gastric wall and inferior displacement of the transverse colon. Additionally, we observed anterior and medial displacement of the inferior mesenteric vein. These findings were consistent with a left paraduodenal hernia causing small bowel obstruction (Fig. 2). While there were no definitive radiological features of bowel strangulation at this time, the clinical presentation and imaging findings raised significant concern for this possibility.

Based on the clinical presentation and imaging findings, we diagnosed a left paraduodenal hernia with small bowel obstruction and suspected strangulation. Given the acute presentation and high risk for bowel compromise, we planned immediate emergency laparoscopic surgery.

## 3 Surgical technique

We placed the patient in the supine position under general anesthesia. We employed a four-port technique: a 12-mm camera port at the umbilicus, two 5-mm working ports in the right upper and lower quadrants, and a 5-mm assistant port in the left lower quadrant.

**Fig. 3:**
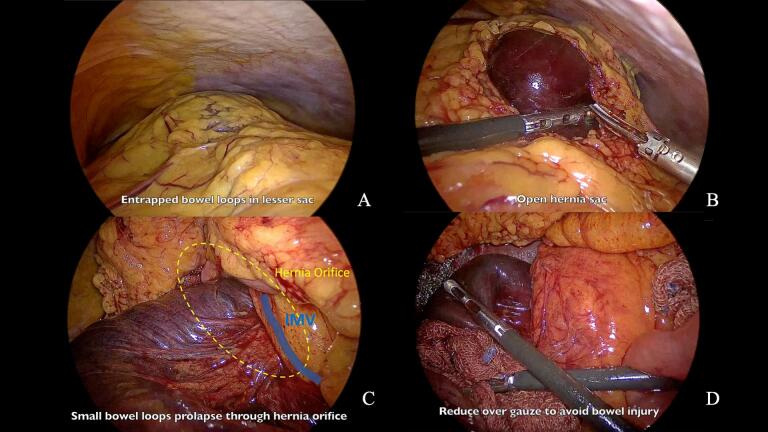
Intraoperative laparoscopic views of left paraduodenal hernia. A: Entrapped small bowel loops in lesser sac, B: Open hernia sac exposing strangulated bowel, C: Left paraduodenal hernia with the inferior mesenteric vein visible at the anterior margin, D: Hernia reduction. Sequential laparoscopic views demonstrating (A) entrapped bowel loops, (B) opened hernia sac revealing strangulated bowel, (C) anatomical landmarks including the inferior mesenteric vein, and (D) process of hernia reduction.

Initial laparoscopic exploration confirmed the presence of entrapped small bowel loops within the lesser sac (Fig. 3A). Critically, segments of small bowel showed signs of ischemia (Fig. 3B), confirming our suspicion of bowel strangulation. Serosanguinous fluid was present in the peritoneal cavity. We identified the inferior mesenteric vein along the anterior margin of the hernia neck (Fig. 3C).

**Fig. 4:**
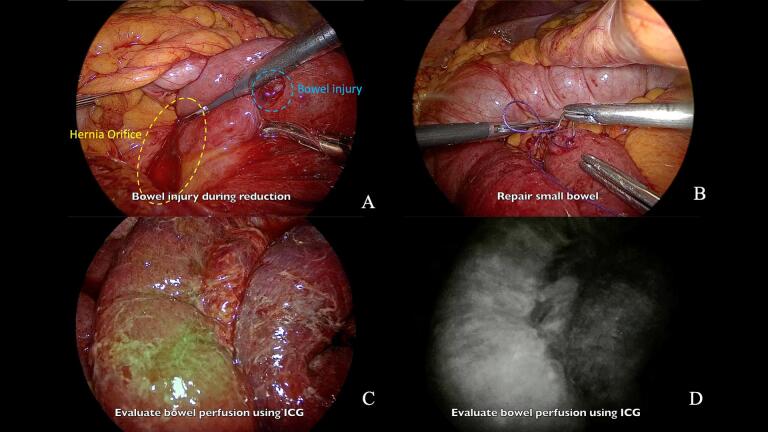
Small bowel injury management and ICG fluorescence imaging. A: Small bowel injury during reduction, B: Repair of small bowel, C: ICG fluorescence imaging (Overlay mode), D: ICG fluorescence imaging (Monochromatic mode). (A) Iatrogenic small bowel injury during hernia reduction, (B) laparoscopic repair of the injury, (C,D) ICG fluorescence imaging in overlay and monochromatic modes for assessment of bowel perfusion.

Reduction of the incarcerated bowel proved challenging due to significant dilatation, friability, and edema of the herniated bowel wall. To facilitate reduction, we carefully opened the hernia sac using an ultrasonic energy device to release pressure (Fig. 3B). We then gently reduced the bowel over gauze to prevent injury (Fig. 3D). Despite these precautions, a small bowel injury occurred during the reduction process (Fig. 4A), which we immediately repaired with interrupted 3–0 Vicryl sutures (Fig. 4B).

Following complete reduction, we used indocyanine green (ICG) fluorescence imaging to assess bowel perfusion and guide resection. We administered a dose of 0.2 mg/kg of ICG intravenously and performed imaging using a near-infrared camera system. This real-time perfusion assessment was crucial in identifying the extent of bowel ischemia and precisely delineating the segment of non-viable bowel requiring resection (Fig. 4C, D).

**Fig. 5:**
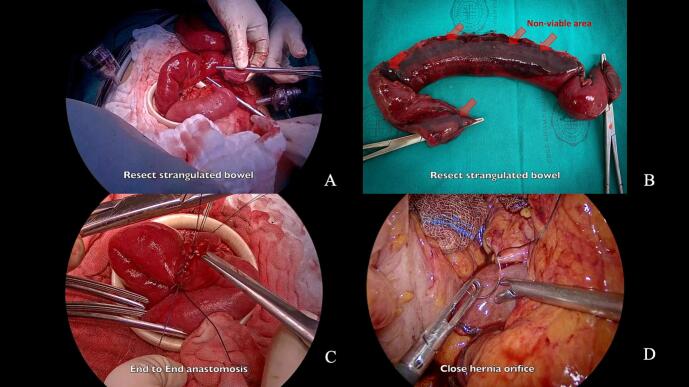
Bowel resection, anastomosis, and hernia defect closure. A: Small bowel resection, B: Resected bowel specimen, C: Bowel anastomosis, D: Closure of hernia orifice. (A) Exteriorization and resection of non-viable bowel segment, (B) resected bowel specimen showing ischemic changes, (C) completed hand-sewn end-to-end anastomosis, (D) closure of the hernia defect using non-absorbable sutures.

We extended the camera port incision to 4 cm, applied a wound protector, and exteriorized the affected bowel segment (Fig. 5A). Before proceeding with the resection, we repeated ICG imaging to confirm the margins of the non-viable segment, ensuring we preserved as much viable bowel as possible. After resection (Fig. 5B), prior to anastomosis, we again utilized ICG imaging to confirm adequate perfusion of the bowel ends. We then performed an end-to-end anastomosis using a hand-sewn technique with 3–0 Vicryl sutures (Fig. 5C).

Next, we closed the hernia defect using four interrupted 2–0 non-absorbable sutures between the peritoneum alongside the inferior mesenteric vein and the duodenal wall (Fig. 5D). We placed a drain in proximity to the anastomosis site to monitor for potential leakage.

The total operative time was 150 min with minimal blood loss (<50 mL). We encountered no further intraoperative complications. A video 1 demonstrating key aspects of the procedure is available as supplementary material.

## 4 Postoperative course

The patient's postoperative recovery was uneventful, reflecting the benefits of the minimally invasive approach despite the complexity of the case. She tolerated oral intake on postoperative day 3 and was discharged home on day 5. At the 8-week follow-up, the patient had returned to normal activities with a well-healed surgical scar. A subsequent follow-up at 9 months post-surgery found the patient to be entirely asymptomatic, with no signs of recurrence or complications.

## 5 Discussion

This case report presents the successful laparoscopic management of a left paraduodenal hernia complicated by small bowel obstruction and strangulation in a 56-year-old woman. Several aspects of this case merit further discussion in the context of current literature and minimally invasive approaches to managing strangulated internal hernias.

## 6 Diagnostic challenges in strangulated paraduodenal hernias

Paraduodenal hernias often present a diagnostic challenge due to their rarity and variable clinical presentation [10]. In our case, the acute onset of symptoms led to prompt imaging, which was crucial in establishing the diagnosis. The CT findings of clustered bowel loops between the stomach and pancreas, with displacement of surrounding structures, are characteristic of left paraduodenal hernias [11,12]. However, the radiological diagnosis of bowel strangulation remains challenging. This case underscores the importance of considering rare anatomical anomalies in the differential diagnosis of small bowel obstruction, especially in patients without previous abdominal surgery [13], and maintaining a high index of suspicion for strangulation based on clinical presentation.

Recent advances in CT technology have further improved the accuracy of preoperative diagnosis. Dilauro et al. (2017) described optimal CT signs for diagnosing internal hernias, including the “swirl sign” and mesenteric vessel abnormalities, which we observed in our case [7]. Doishita et al. (2016) emphasized the importance of multiplanar reformations in identifying the hernia orifice and contents [14]. These imaging advancements have significantly contributed to earlier and more accurate diagnosis of paraduodenal hernias, though the definitive assessment of bowel viability often remains an intraoperative finding.

Laparoscopic Approach in Emergency Settings with Bowel Strangulation.

While laparoscopic repair of paraduodenal hernias has been reported in elective settings [15], its application in emergency scenarios with bowel obstruction and strangulation is less common. Our case demonstrates the feasibility and safety of the laparoscopic approach even in acute presentations with compromised bowel. This aligns with recent literature suggesting that laparoscopy can be successfully employed in select cases of complicated paraduodenal hernias [4].

Wakabayashi et al. (2018) reported a similar case of successful laparoscopic repair of an acute left paraduodenal hernia, highlighting the importance of careful bowel manipulation and systematic reduction techniques [16]. Somuncu and Bozdag (2022) also demonstrated the feasibility of the laparoscopic approach in an emergency setting, emphasizing the importance of proper port placement for optimal visualization and manipulation [17]. Our four-port technique provided excellent visualization and maneuverability, crucial for managing the strangulated bowel safely.

The benefits of minimally invasive surgery, including faster recovery and reduced postoperative pain, were evident in our patient's uneventful recovery and short hospital stay, despite the presence of bowel strangulation. This is consistent with the findings of Jeong et al. (2008), who reported shorter hospital stays and faster return to normal activities in patients undergoing laparoscopic repair compared to open surgery [12].

## 7 Management of intraoperative complications in strangulated hernias

The case highlights the potential challenges in reducing incarcerated and strangulated bowel laparoscopically. Despite careful manipulation, bowel injury occurred during reduction, necessitating immediate repair. This complication underscores the importance of meticulous technique and readiness to address unexpected issues during laparoscopic hernia reduction, particularly in cases of bowel strangulation [16,18]. Gentle traction and careful dissection are crucial to avoid further injury to compromised bowel. In cases of difficult reduction, opening the hernia sac may facilitate the process [16,17], a technique we employed successfully in our case. The successful management of this complication without conversion to open surgery further demonstrates the versatility of the laparoscopic approach, even in complex strangulated hernias.

## 8 Use of ICG fluorescence imaging in assessing bowel viability

A notable aspect of this case was the use of indocyanine green (ICG) fluorescence imaging to assess bowel perfusion in the setting of strangulation. This technology, while increasingly used in colorectal surgery, has not been widely reported in the context of paraduodenal hernia repair with bowel ischemia [19,20]. ICG imaging provided real-time assessment of bowel viability, guiding our decision for bowel resection and ensuring adequate perfusion prior to anastomosis [21]. This technique represents a significant advancement over traditional visual assessment, particularly in cases of questionable bowel viability.

The use of ICG fluorescence imaging in emergency hernia surgery with suspected bowel ischemia has been previously reported. Gianchandani Moorjani et al. (2021) described its application in evaluating intestinal viability during laparoscopic repair of an incarcerated hernia [22]. Our case further demonstrates the utility of this technique in complex emergency scenarios involving paraduodenal hernias with strangulation.

Recent literature has shown the increasing utility of ICG in various gastrointestinal procedures involving compromised blood supply. Belia et al. (2022) reviewed the use of ICG in gastric cancer surgery, demonstrating its value in assessing perfusion and lymph node mapping [23]. Garoufalia and Wexner (2023) highlighted the potential of ICG-guided surgery in reducing anastomotic leaks in colorectal procedures [24]. Our case suggests that ICG imaging could be a valuable tool in the management of complicated paraduodenal hernias with strangulation, potentially reducing the risk of missed bowel ischemia and subsequent anastomotic complications.

The utility of ICG in assessing bowel viability extends beyond hernia repairs. Karampinis et al. (2018) demonstrated that ICG tissue angiography could reduce the extent of bowel resections in acute mesenteric ischemia [25]. This further supports the potential of ICG imaging in guiding surgical decision-making in various acute abdominal conditions involving compromised bowel perfusion.

## 9 Hernia defect closure techniques

The approach to hernia defect closure in paraduodenal hernias typically favors primary repair, with mesh repair reserved only for exceptionally large defects [26]. In our case, we found that the defect could be adequately closed with primary sutures. This decision aligns with the majority of reported cases in the literature, including the approach described by Khalaileh et al. (2010) in their report of left laparoscopic paraduodenal hernia repair [13]. The technique we used for closing the hernia defect with interrupted sutures between the peritoneum along the inferior mesenteric vein and the duodenal wall is consistent with methods described in most case reports and series [27]. While C. Palanivelu et al. have reported using mesh for large defects and recurrent hernia, primary repair remains the preferred method for most paraduodenal hernias when the defect can be closed without tension [26]. Our case further supports the efficacy of primary closure in managing these rare hernias.

## 10 Long-term outcomes after repair of strangulated paraduodenal hernias

The favorable outcome in this case, with complete symptom resolution and no recurrence at 9 months, is encouraging, especially considering the complexity of the initial presentation with bowel strangulation. However, long-term follow-up data on laparoscopically repaired paraduodenal hernias, particularly those involving bowel resection, are limited in the literature [28]. A systematic review by Schizas et al. (2019) analyzed 166 cases of paraduodenal hernias, including 59 managed laparoscopically [8]. They reported low recurrence rates and excellent long-term outcomes for laparoscopic repairs, supporting the efficacy of this approach. However, their review did not specifically focus on cases with bowel strangulation.

This case contributes to the growing body of evidence supporting laparoscopic management of complicated paraduodenal hernias but also highlights the need for longer-term studies to fully assess the durability of repair and potential for late complications, especially in cases involving bowel resection [8]. Future research should focus on the long-term outcomes of patients who undergo emergency repair for strangulated paraduodenal hernias, including the potential for adhesive small bowel obstruction or recurrence.

## 11 Conclusion

This case demonstrates that emergency laparoscopic management of complicated left paraduodenal hernias with bowel strangulation is feasible and can lead to excellent outcomes. The use of advanced imaging techniques like ICG fluorescence may enhance intraoperative decision-making, particularly in assessing bowel viability in cases of suspected strangulation. This approach potentially reduces the extent of bowel resection and improves outcomes in these complex cases.

As experience with laparoscopic approaches to these rare hernias grows, further refinement of techniques and long-term outcome data will be valuable in establishing best practices for management. Future research should focus on standardizing laparoscopic techniques for managing strangulated paraduodenal hernias, evaluating long-term outcomes after bowel resection, and exploring the role of novel technologies like ICG imaging in improving surgical outcomes for these challenging cases.

The following is the supplementary data related to this article.

Supplementary video 1 Laparoscopic Management of Obstructed Left Paraduodenal Hernia with ICG Fluorescence Imaging. Supplementary video 1

## Patient consent

Written informed consent was obtained from the patient for publication and any accompanying images. A copy of the written consent is available for review by the Editor-in-Chief of this journal on request.

## Ethical approval

Ethical approval was not required for this case report as per our institutional guidelines.

## Guarantor

Hariruk Yodying accepts full responsibility for the work, had access to the data, and controlled the decision to publish.

## Research registration number

1. Name of the registry: Research Registry.
2. Unique identifying number or registration ID: researchregistry10740.
3. Hyperlink to your specific registration (must be publicly accessible and will be checked): https://www.researchregistry.com/browse-the-registry#home/.

## Funding

This research did not receive any specific grant from funding agencies in the public, commercial, or not-for-profit sectors.

## Author contribution

Hariruk Yodying: Conceptualization, data collection, writing - original draft, review and editing.

## Conflict of interest statement

The authors declare that they have no known competing financial interests or personal relationships that could have appeared to influence the work reported in this paper.
